# The neutrophil percentage-to-albumin ratio is associated with all-cause mortality in critically ill patients with acute myocardial infarction

**DOI:** 10.1186/s12872-022-02559-z

**Published:** 2022-03-18

**Authors:** Ya Lin, Yanhan Lin, Juanqing Yue, Qianqian Zou

**Affiliations:** 1 Department of Cardiology, The First Affiliated Hospital of Wenzhou Medical University, Wenzhou, 325000 Zhejiang China; 2 Department of Pathology, Affiliated Hangzhou First People’s Hospital, Zhejiang University School of Medicine, Hangzhou, 310000 Zhejiang China; 3 Obstetrics and Gynecology Ultrasonic Department, Wenzhou People’s Hospital, Wenzhou, 325000 Zhejiang China

**Keywords:** Neutrophil percentage-to-albumin ratio, All-cause mortality, Acute myocardial infarction

## Abstract

**Aim:**

In this study, we evaluated the utility of neutrophil percentage-to-albumin ratio (NPAR) in predicting in critically ill patients with acute myocardial infarction (AMI).

**Methods:**

The information of patients were collected from Medical Information Mart for Intensive Care III database. Admission NPAR was calculated as neutrophil percentage divided by serum albumin. The endpoints of this study were 30-day, 90-day, 180-day, and 365-day all-cause mortality. Cox proportional hazards models and subgroup analyses were used to determine the relationship between admission NPAR and these endpoints.

**Results:**

798 critically ill patients with AMI were enrolled in. After adjustments for age, race and gender, higher admission NPAR was associated with increased risk of 30-day, 90-day, 180-day, and 365-day all-cause mortality in critically ill patients with AMI. And after adjusting for possible confounding variables, two different trends have emerged. Stratified by tertiles, high admission NPAR was independently associated with 180-day and 365-day all-cause mortality in critically ill patients with AMI (tertile 3 vs. tertile 1: adjusted HR, 95% CI 1.71, 1.10–2.66, *p* < 0.05; 1.66, 1.10–2.51, *p* < 0.05). In other hand, stratified by quartiles, highest admission NPAR levels were independently associated with 90-day, 180-day and 365-day all-cause mortality (quartile 4 vs. quartile 1: adjusted HR, 95% CI 2.36, 1.32–4.23, *p* < 0.05; 2.58, 1.49–4.47, *p* < 0.05; 2.61, 1.56–4.37, *p* < 0.05). ROC test showed that admission NPAR had a moderate ability to predict all-cause mortality of critically ill patients with AMI. No obvious interaction was found by subgroup analysis in most subgroups.

**Conclusions:**

Admission NPAR was an independent predictor for 180-day and 365-day all-cause mortality in critically ill patients with AMI.

## Introduction

As it is well known, acute myocardial infarction (AMI) as a common cardiovascular disease continues to be the leading cause of hospital admission and mortality rate worldwide in the past years [1]. Although potent antiplatelet agents and early revascularization has greatly reduced the occurrence of major adverse cardiovascular events in AMI patients [2], the prognosis of AMI patients is still an apparent problem worthy of our attention.

The inflammatory response participates in myocardial infarction (MI) area and left ventricular (LV) remodeling [3, 4]. Neutrophil, the major participant in the inflammatory burst, mediates the inflammatory response to myocardial injury [3, 5]. Albumin acts through its multiple binding sites and free radical-capture properties, involved in antioxidant activities [6]. It has been confirmed that low albumin level has a significant effect on the mortality and prognosis of AMI [7–9].

According to the previous studies, admission neutrophil percentage-to-albumin ratio (NPAR) was an independent predictor of in-hospital mortality in patients with acute ST-segment elevation myocardial infarction (STEMI) [10]. In the another study, in critically ill patients with coronary artery disease (CAD), the higher NPAR level was closely correlated with the higher rate of 30-day, 90-day, and 365-day all-cause death [11]. However, there is no study yet that reported the association between admission NPAR level and the all-cause mortality in critically ill patients with AMI. For all the above reasons, in this study we hypothesized that admission NPAR level could be a prognostic predictor of all-cause mortality in critically ill patients with AMI.

## Method

### Source of data

We conducted a retrospective cohort study, where the data was collected from a large, single-center critical care database called Medical Information Mart for Intensive Care III (MIMIC III) v1.4 [12]. MIMIC III is a public and freely available database and integrates comprehensive clinical data of patients admitted to the intensive care units (ICU) at the Beth Israel Deaconess Medical Center between 2001 and 2012. This database was approved by the Institutional Review Boards (IRB) of the Massachusetts Institute of Technology (MIT). After successfully completing the National Institutes of Health (NIH) Web-based training course and the Protecting Human Research Participants examination (no. 40683764), we were given the permission to extract data from MIMIC III.

### Population selection criteria

All ICU inpatients with AMI diagnosed by ICD-9 diagnosis code were enrolled in this study. Exclusion criteria were as follows: (1) multiple ICU admissions; (2) aged < 18 years old; (3) had died before admission; (4) lack of the information of albumin and neutrophil percentage during ICU stay.

### Data extraction and definition of NPAR

Data extraction from MIMIC III was accomplished using Structured Query Language (SQL) with the PostgreSQL tool (version 9.6). We extracted demographics, vital signs, comorbidities and medical history, laboratory parameters, scoring system, medication use, and clinical survival information. Age, gender and race were contained in demographics, and vital signs included heart rate, respiratory rate and mean blood pressure (MBP). Comorbidities and medical history included CAD, prior MI, atrial fibrillation (AF), chronic heart failure (CHF), chronic kidney disease (CDK), chronic obstructive pulmonary disease (COPD) and percutaneous transluminal coronary angioplasty/percutaneous coronary intervention (PTCA/PCI). Laboratory parameters were consisted of neutrophils, albumin, hemoglobin, white blood cell (WBC), prothrombin time (PT), platelet, red cell distribution width (RDW), potassium, sodium, creatinine, blood urea nitrogen (BUN), alanine aminotransferase (ALT), aspartate aminotransferase (AST), creatine kinase-peak (CK), MB isoenzyme of creatine kinase (CK-MB), and glucose. Scoring systems contains two important scoring scales—sequential organ failure assessment (SOFA) score [13] and simplified acute physiology score II (SAPS II) [14]. In addition, the medication use of patients in this study would be shown in the baseline characteristics, including whether they have used aspirin, clopidogrel, metoprolol, angiotensin-converting enzyme inhibitors/angiotensin receptor blockers (ACEI/ARBs) or statins. The other extracted data have weight and urine output within 24 h. All the laboratory parameters were firstly-measured data after admission to the ICU. The endpoints of this study were 30-day, 90-day, 180-day, and 365-day all-cause mortality. We divided neutrophil percentage by albumin to get admission NPAR [10].

### Statistical analysis

All the patients with AMI in the study were stratified in term of admission NPAR tertiles. Data distribution of all continuous variables were tested using the Shapiro–Wilk test, and they were nonnormally distributed, and manifested as median and interquartile range (Q1–Q3). All categorical data was expressed as number and percentage. Kruskal–Wallis or Fisher's exact test was performed to evaluate statistical differences among different groups of NPAR.

By using Log-rank tests, survival rates of different groups were compared, and the Kaplan–Meier curves were built.

In order to evaluate the independent effect of admission NPAR on 30-day, 90-day, 180-day, and 365-day all-cause mortality, cox proportional hazard models were developed. The first tertile and quartile groups of admission NPAR were treated as the reference group, and the results were summarized as hazard ratios (HR) with 95% confidence intervals (CI). In model I, age, race and gender were incorporated into adjustment. In model II, we further adjusted for age, gender, race, respiratory rate, MBP, heart rate, ALT, AST, CK-peak, CK-MB-peak, glucose, PT, hemoglobin, RDW, creatinine, potassium, sodium, BUN, WBC, platelet, CAD, AF, COPD, hypertension, diabetes, prior MI, CHF, CKD, stroke, SOFA and SAPS II. And P for trend was calculated. Subgroup analysis was conducted to estimate the effect of admission NPAR on 180-day all-cause mortality. And we got the *P* value for interaction.

Receiver-operating characteristic (ROC) curve was performed to measure the sensitivity and specificity of admission NPAR, as well as SOFA score. Moreover, the area under the curve (AUC) was calculated to estimate the quality of admission NPAR as a predictor of 365-day all-cause mortality. Statistical analyses were performed using EmpowerStats version 2.0 (http://www.empowerstats.com/cn/, X&Y solutions, Inc., Boston, MA) and R software version 3.4.3; *P* value of < 0.05 was considered to be statistically significant.

## Result

### Baseline characteristics of patients

**Fig. 1:**
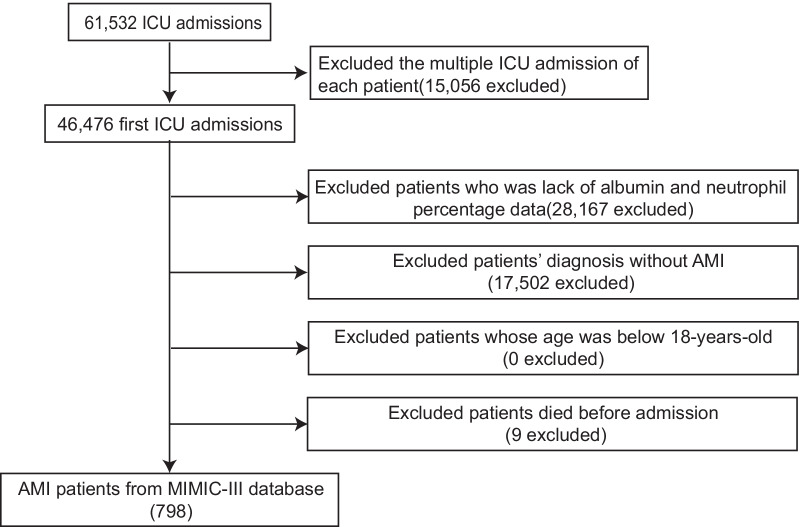
Flow chat of study selection. *ICU* intensive care unit, *AMI* acute myocardial infarction, *MIMIC III* Medical Information Mart for Intensive Care III

After reviewing the data of 61,532 critically ill patients, a total of 798 patients with AMI were enrolled in our study (Fig. 1). Based on tertiles of admission NPAR level, participants were categorized into three groups (tertile 1: < 21.58; tertile 2: ≥ 21.58, < 26.77; and tertile 3: ≥ 26.77), and each group included 266 AMI patients. The baseline characteristics were displayed in Table 1. Patients in the highest tertile of admission NPAR level were older than other groups, and most of them were white. In addition, they reported more medical history of AF, but less comorbidities of CAD, hypertension and CHF. Moreover, patients in the highest tertile of admission NPAR level were less likely to use aspirin, clopidogrel, metoprolol, ACEI/ARBs and statin, and to receive PTCA or PCI. Finally, they had lower MBP, weight, albumin, hemoglobin, urine output in 24 h, and higher values of heart rate, neutrophils, WBC, PT, RDW, creatinine, BUN, ALT, AST, SAPS II and SOFA.

**Table 1:** Characteristics of study patients by NPAR tertiles

Characteristics	Tertile 1 (n = 266) < 21.58	Tertile 2 (n = 266) ≥ 21.58, < 26.77	Tertile 3 (n = 266) ≥ 26.77	*p* value
Age (years)	67.50 (57.00–77.75)	69.00 (58.00–79.00)	73.00 (63.00–80.00)	0.001
Male, n (%)	179 (67.29%)	165 (62.03%)	160 (60.15%)	0.209
*Race, n (%)*				0.032
White	179 (67.29%)	160 (60.15%)	179 (67.29%)	
Black	21 (7.89%)	17 (6.39%)	8 (3.01%)	
Other	66 (24.81%)	89 (33.46%)	79 (29.70%)	
Heart rate (beats/minute)	95.77 ± 25.54	101.57 ± 26.39	105.51 ± 24.40	< 0.001
MBP (mmHg)	73.15 ± 34.18	67.20 ± 28.90	62.95 ± 29.72	< 0.001
Respiratory rate (beats/minute)	24.24 ± 8.82	24.33 ± 9.81	25.47 ± 9.94	0.240
Weight (kg)	81.94 ± 19.31	79.13 ± 19.65	77.97 ± 19.72	0.017
CAD	192 (72.18%)	207 (77.82%)	146 (54.89%)	< 0.001
Prior MI	15 (5.64%)	18 (6.77%)	13 (4.89%)	0.645
AF	67 (25.19%)	80 (30.08%)	100 (37.59%)	0.008
Hypertension	136 (51.13%)	118 (44.36%)	91 (34.21%)	< 0.001
Diabetes	75 (28.20%)	76 (28.57%)	80 (30.08%)	0.880
CHF	105 (39.47%)	136 (51.13%)	135 (50.75%)	0.009
CKD	29 (10.90%)	37 (13.91%)	42 (15.79%)	0.251
Stroke	6 (2.26%)	10 (3.76%)	14 (5.26%)	0.190
COPD	4 (1.50%)	2 (0.75%)	8 (3.01%)	0.131
PTCA/PCI	187 (70.30%)	173 (65.04%)	125 (46.99%)	< 0.001
*Laboratory parameters*				
Neutrophil percentage (%)	71.20 (61.35–78.95)	84.00 (77.60–88.07)	86.00 (80.85–89.77)	< 0.001
Albumin (g/dL)	3.80 (3.50–4.10)	3.50 (3.20–3.70)	2.70 (2.40–3.00)	< 0.001
Hemoglobin (g/dL)	13.35 (11.90–14.57)	12.60 (11.10–14.10)	11.35 (10.10–12.97)	< 0.001
WBC (10^9^/L)	10.30 (7.50–13.80)	12.50 (9.60–16.70)	13.70 (10.10–17.50)	< 0.001
PT (seconds)	13.30 (12.50–14.00)	13.70 (12.90–15.10)	14.50 (13.30–16.88)	< 0.001
Platelet (10^9^/L)	242.50 (188.25–301.75)	252.00 (192.25–299.00)	230.50 (170.25–302.75)	0.113
RDW (%)	13.60 (13.10–14.50)	13.70 (13.00–14.50)	14.20 (13.30–15.60)	< 0.001
Potassium (mmol/L)	4.20 (3.90–4.60)	4.10 (3.80–4.50)	4.20 (3.80–4.70)	0.216
Sodium (mmol/L)	139.00 (137.00–141.00)	138.00 (135.25–141.00)	139.00 (134.25–141.00)	0.036
Creatinine (mEq/L)	1.10 (0.90–1.40)	1.10 (0.90–1.40)	1.20 (1.00–1.70)	< 0.001
BUN (mg/dL)	20.00 (15.00–28.00)	21.00 (15.00–30.25)	27.00 (18.50–43.50)	< 0.001
ALT (U/L)	30.21 (20.00–62.75)	37.00 (21.00–68.75)	42.33 (22.00–79.00)	0.024
AST (U/L)	52.00 (26.25–124.63)	74.50 (34.24–174.00)	77.70 (38.00–196.37)	0.001
CK-peak (U/L)	743.30 (292.70–1756.00)	1088.50 (329.25–2427.20)	702.00 (252.15–1983.00)	0.052
CK-MB-peak (U/L)	53.00 (14.00–181.00)	72.00 (13.00–227.50)	37.00 (11.00–150.00)	0.092
Glucose (mg/dL)	143.00 (115.25–191.75)	148.00 (122.25–207.25)	152.00 (119.25–213.75)	0.254
*Scoring systems*				
SAPS II	34.00 (25.00–44.00)	36.00 (28.00–48.00)	43.00 (35.00–54.00)	< 0.001
SOFA	3.00 (1.00–2.00)	4.00 (3.00–5.00)	6.00 (7.00–10.00)	< 0.001
*Medication use, n (%)*				
Aspirin	214 (80.45%)	221 (83.08%)	172 (64.66%)	< 0.001
Clopidogre	132 (49.62%)	148 (55.64%)	117 (43.98%)	0.027
Metoprolol	209 (78.57%)	197 (74.06%)	156 (58.65%)	< 0.001
ACEI/ARBs	156 (58.65%)	171 (64.29%)	100 (37.59%)	< 0.001
Statin	201 (75.56%)	199 (74.81%)	156 (58.65%)	< 0.001
Urine output (ml/24 h)	2116.00 (1424.00–3074.00)	1825.00 (1090.00–2718.75)	1377.50 (812.25–2275.25)	< 0.001

Continuous variables are presented as mean (SD) for normally distributed variables or median (interquartile range) for non-normally distributed variables, whereas categorical variables are presented as number (percentage)

*MBP* mean blood pressure, *CAD* coronary artery disease, *prior MI* prior myocardial infarction, *AF* atrial fibrillation, *CHF* chronic heart failure, *CDK* chronic kidney disease, *COPD* chronic obstructive pulmonary disease, *PTCA/PCI* percutaneous transluminal coronary angioplasty/percutaneous coronary intervention, *WBC* white blood cell, *PT* prothrombin time, *RDW* red cell distribution width, *BUN* blood urea nitrogen, *ALT* alanine aminotransferase, *AST* aspartate aminotransferase, *CK* creatine kinase, *CK-MB* MB isoenzyme of creatine kinase, *SAPS II* simplified acute physiology score II, *SOFA* sequential organ failure assessment score, *ACEI/ARBs* angiotensin-converting enzyme inhibitor/angiotensin receptor blockers

### Admission NPAR and outcome

As it had been shown in Table 2, the overall length of ICU stay (LOS) was 3.69 days, and the overall in-hospital, 30-day, 90-day, 180-day and 365-day all-cause mortality were 17.92%, 19.67%, 25.44%, 29.82% and 33.21%, respectively. Furthermore, as admission NPAR levels increased, the all-cause death rate of in-hospital, 30-day, 90-day, 180-day and 365-day were distinctly raised.

**Table 2:** Outcome of the study patients by NPAR tertiles

Outcomes	Total (n = 798)	Tertile 1 (n = 266) < 21.58	Tertile 2 (n = 266) ≥ 21.58, < 26.77	Tertile 3 (n = 266) ≥ 26.77	*p* value
ICU LOS (day)	3.69 (0.51–100.12)	2.57 (0.58–49.13)	3.71 (0.59–100.12)	5.33 (0.51–52.81)	< 0.001
*All-cause mortality*					
In-hospital	143 (17.92%)	32 (12.03%)	38 (14.29%)	73 (27.44%)	< 0.001
30-day mortality	157 (19.67%)	35 (13.16%)	48 (18.05%)	74 (27.82%)	< 0.001
90-day mortality	203 (25.44%)	44 (16.54%)	58 (21.80%)	101 (37.97%)	< 0.001
180-day mortality	238 (29.82%)	50 (18.80%)	73 (27.44%)	115 (43.23%)	< 0.001
365-day mortality	265 (33.21%)	58 (21.80%)	84 (31.58%)	123 (46.24%)	< 0.001

Data are expressed as count (percentage) for categorical variables and median (interquartile range) for continuous variables

*ICU LOS* length of ICU stay

**Fig. 2:**
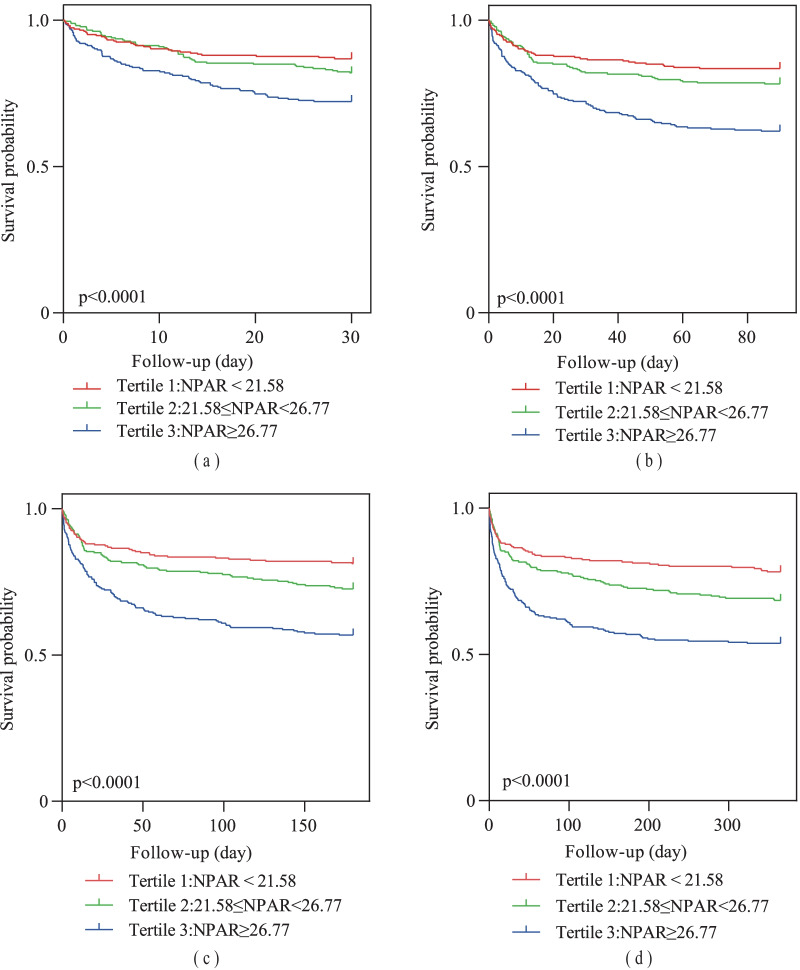
Survival probability and Kaplan–Meier curves of three admission NPAR groups. **a** 30-day. **b** 90-day. **c** 180-day. **d** 365-day. *NPAR* neutrophil percentage-albumin ratio

There were the survival curves of 30-day (log-rank, *p* < 0.0001), 90-day (log-rank, *p* < 0.0001), 180-day (log-rank, *p* < 0.0001) and 365-day (log-rank, *p* < 0.0001) all-cause mortality stratified by the tertiles of admission NPAR, which were manifested in Fig. 2. The trends indicated that the higher NPAR level had a worse survival probability.

### Admission NPAR as a predictor of the clinical endpoints

In cox regression models, admission NPAR levels were stratified by tertiles and quartiles, to appraise whether admission NPAR was related to 30-day, 90-day, 180-day and 365-day all-cause mortality (Table 3). In model I, after adjustments for age, race and gender, higher admission NPAR was associated with increased risk of all-cause mortality. In model II, age, gender, race, respiratory rate, MBP, heart rate, ALT, AST, CK-peak, CK-MB-peak, glucose, PT, hemoglobin, RDW, creatinine, potassium, sodium, BUN, WBC, platelet, CAD, AF, COPD, hypertension, diabetes, prior MI, CHF, CKD, stroke, SOFA and SAPS II were incorporated into the regression model. There was a prominent correlation between high admission NPAR, 180-day and 365-day all-cause mortality (tertile 3 vs. tertile 1: adjusted HR, 95% CI 1.71, 1.10–2.66, *p* < 0.05; 1.66, 1.10–2.51, *p* < 0.05). However, the relationship between admission NPAR, 30-day and 90-day all-cause mortality was not as relevant as the other groups. Unexpectedly, a merely different trend was observed in admission NPAR levels stratified by quartiles; highest admission NPAR levels were independently associated with 90-day, 180-day and 365-day all-cause mortality (quartile 4 vs. quartile 1: adjusted HR, 95% CI 2.36, 1.32–4.23; 2.58, 1.49–4.47; 2.61, 1.56–4.37, *p* < 0.05).

**Table 3:** The association between all-cause mortality and NPAR

NPAR	Non-adjusted		Model I		Model II	
	HR (95% CIs)	*p* value	HR (95% CIs)	*p* value	HR (95% CIs)	*p* value
*30-day all-cause mortality*						
*Tertiles*						
< 21.58	1.0		1.0		1.0	
≥ 21.58, < 26.77	1.38 (0.89, 2.13)	0.1506	1.27 (0.82, 1.96)	0.2917	0.99 (0.60, 1.66)	0.9826
≥ 26.77	2.29 (1.53, 3.43)	< 0.0001	2.07 (1.38, 3.12)	0.0005	1.29 (0.77, 2.17)	0.3262
P for trend	< 0.0001		0.0002		0.5418	
*Quartiles*						
< 20.49	1.0		1.0		1.0	
≥ 20.49, < 23.94	1.55 (0.91, 2.65)	0.1078	1.43 (0.84, 2.45)	0.1912	1.73 (0.91, 3.30)	0.0956
≥ 23.94, < 28.33	2.07 (1.24, 3.45)	0.0056	1.96 (1.17, 3.28)	0.0109	1.50 (0.80, 2.83)	0.2101
≥ 28.33	2.87 (1.76, 4.69)	< 0.0001	2.54 (1.54, 4.18)	0.0002	2.01 (1.05, 3.87)	0.0354
P for trend	< 0.0001		< 0.0001		0.2698	
*90-day all-cause mortality*						
*Tertiles*						
< 21.58	1.0		1.0		1.0	
≥ 21.58, < 26.77	1.34 (0.91, 1.98)	0.1439	1.22 (0.82, 1.81)	0.3200	1.06 (0.66, 1.70)	0.7952
≥ 26.77	2.59 (1.81, 3.68)	< 0.0001	2.31 (1.61, 3.31)	< 0.0001	1.54 (0.97, 2.46)	0.0701
P for trend	< 0.0001		< 0.0001		0.0893	
*Quartiles*						
< 20.49	1.0		1.0		1.0	
≥ 20.49, < 23.94	1.60 (1.00, 2.58)	0.0504	1.48 (0.92, 2.38)	0.1077	1.86 (1.04, 3.33)	0.0359
≥ 23.94, < 28.33	1.85 (1.16, 2.94)	0.0099	1.75 (1.09, 2.79)	0.0195	1.41 (0.78, 2.53)	0.2512
≥ 28.33	3.39 (2.21, 5.20)	< 0.0001	2.97 (1.92, 4.58)	< 0.0001	2.36 (1.32, 4.23)	0.0038
P for trend	< 0.0001		< 0.0001		0.0399	
*180-day all-cause mortality*						
*Tertiles*						
< 21.58	1.0		1.0		1.0	
≥ 21.58, < 26.77	1.50 (1.05, 2.15)	0.0273	1.38 (0.96, 1.98)	0.0821	1.36 (0.88, 2.11)	0.1660
≥ 26.77	2.67 (1.92, 3.73)	< 0.0001	2.40 (1.71, 3.36)	< 0.0001	1.71 (1.10, 2.66)	0.0165
P for trend	< 0.0001		< 0.0001		0.0272	
*Quartiles*						
< 20.49	1.0		1.0		1.0	
≥ 20.49, < 23.94	1.78 (1.15, 2.76)	0.0092	1.66 (1.07, 2.57)	0.0236	2.18 (1.27, 3.75)	0.0048
≥ 23.94, < 28.33	1.91 (1.24, 2.95)	0.0036	1.80 (1.17, 2.79)	0.0080	1.66 (0.96, 2.88)	0.0698
≥ 28.33	3.54 (2.37, 5.29)	< 0.0001	3.12 (2.08, 4.68)	< 0.0001	2.58 (1.49, 4.47)	0.0007
P for trend	< 0.0001		< 0.0001		0.0150	
*365-day all-cause mortality*						
*Tertiles*						
< 21.58	1.0		1.0		1.0	
≥ 21.58, < 26.77	1.50 (1.08, 2.10)	0.0169	1.38 (0.99, 1.93)	0.0610	1.40 (0.93, 2.10)	0.1031
≥ 26.77	2.52 (1.85, 3.45)	< 0.0001	2.22 (1.62, 3.04)	< 0.0001	1.66 (1.10, 2.51)	0.0154
P for trend	< 0.0001		< 0.0001		0.0471	
*Quartiles*						
< 20.49	1.0		1.0		1.0	
≥ 20.49, < 23.94	1.76 (1.17, 2.65)	0.0064	1.64 (1.09, 2.46)	0.0182	2.20 (1.32, 3.66)	0.0024
≥ 23.94, < 28.33	1.91 (1.27, 2.86)	0.0018	1.78 (1.18, 2.67)	0.0056	1.80 (1.08, 3.00)	0.0250
≥ 28.33	3.39 (2.33, 4.95)	< 0.0001	2.92 (2.00, 4.28)	< 0.0001	2.61 (1.56, 4.37)	0.0003
P for trend	< 0.0001		< 0.0001		0.0075	

HR: hazard ratio; CI: Confidence interval. Models were derived from Cox proportional hazard regression models

Non-adjusted model adjust for: None

Adjust I model adjust for: age; gender; race

Adjust II model adjust for: Age; gender; race; respiratory rate; MBP; heart rate; ALT; AST; CK-peak; CK-MB-peak; glucose; PT; hemoglobin; RDW; creatinine; potassium; sodium; BUN; WBC; Platelet; CAD; AF; COPD; hypertension; diabetes; prior MI; CHF; CKD; STROKE; SOFA; SAPS II

**Fig. 3:**
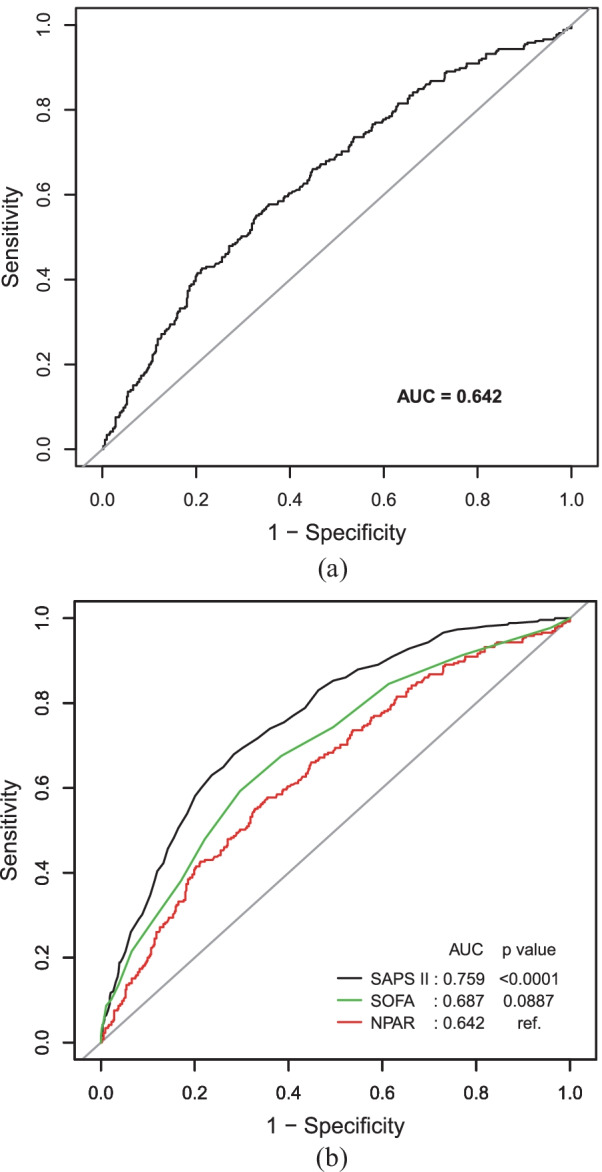
ROC curves for the prediction of 365-day all-cause mortality. **a** About NPAR. **b** About NPAR, SAPS II score, and SOFA score. *NPAR* neutrophil percentage-albumin ratio, *SAPS II* simplified acute physiology score II, *SOFA* sequential organ failure assessment score

The ROC test was employed to measure the sensitivity and specificity of admission NPAR with an AUC of 0.6421 (95% CI 0.6016–0.6826, *p* < 0.0001). Then the AUC area of admission NPAR was compared with SAPS II and SOFA score. There was no difference between NPAR and SOFA. Thus, it ascertained the quality of NPAR as a reliable predictor of 365-day all-cause mortality (Fig. 3).

Then admission NPAR was integrate into SAPS II and SOFA score. The ROC test was performed on the two combined models, and the results obtained were compared with the original models. For a more comprehensive evaluation of the effects of the new models, integrated discrimination improvement (IDI) was introduced. According to Table 4, after adding NPAR, the AUC of the combined models of SOFA score was increased to 0.714 (*p* < 0.001) and the integrated improvement was 4.72%. However, the discrimination between SAPS II and its integrated model is not obvious.

**Table 4:** Discrepancies for the prediction of 365-day all-cause mortality

	AUC	*p* value	IDI	*p* value
SAPS II	0.759	0.113	Ref	0.0736
SAPS II + NPAR	0.769		0.0358	
SOFA	0.687	< 0.001	Ref	< 0.05
SOFA + NPAR	0.714		0.0472	

*AUC* the area under the curve, *IDI* integrated discrimination improvement, *SAPS II* simplified acute physiology score II, *NPAR* neutrophil percentage-to-albumin ratio, *SOFA* sequential organ failure assessment score

### Subgroup analysis

In most subgroups, no significant interaction between admission NPAR and 180-day all-cause mortality was observed (Table 5). Patients with high values of heart rate SAPS II, ICU LOS and age had higher risks of all-cause mortality for high admission NPAR.

**Table 5:** The association between three NPAR groups and 180-day all-cause mortality in subgroup analysis

	N	NPAR < 21.58 (reference)	21.58 ≤ NPAR < 26.77 HR (95% CIs)	NPAR ≥ 26.77 HR (95% CIs)	*p* for interaction
*AF*					0.4736
No	551	1.0	1.42 (0.90, 2.23)	2.80 (1.85, 4.26)	
Yes	247	1.0	1.55 (0.86, 2.81)	2.19 (1.26, 3.82)	
*CHF*					0.1939
No	422	1.0	1.18 (0.68, 2.07)	2.89 (1.79, 4.67)	
Yes	376	1.0	1.60 (0.99, 2.59)	2.30 (1.45, 3.65)	
*CKD*					0.5369
No	690	1.0	1.54 (1.04, 2.27)	2.53 (1.76, 3.64)	
Yes	108	1.0	1.21 (0.47, 3.13)	3.12 (1.35, 7.22)	
*COPD*					0.9520
No	784	1.0	1.50 (1.04, 2.16)	2.62 (1.87, 3.67)	
Yes	14	1.0	2.42 (0.15, 39.05)	3.37 (0.40, 28.33)	
*CAD*					0.8694
No	253	1.0	1.72 (0.92, 3.23)	2.54 (1.48, 4.36)	
Yes	545	1.0	1.45 (0.94, 2.26)	2.49 (1.61, 3.83)	
*Hypertension*					0.3855
No	453	1.0	1.19 (0.74, 1.91)	2.34 (1.54, 3.55)	
Yes	345	1.0	1.95 (1.11, 3.41)	2.86 (1.64, 4.98)	
*Diabetes*					0.3603
No	567	1.0	1.36 (0.90, 2.06)	2.32 (1.58, 3.40)	
Yes	231	1.0	1.99 (0.96, 4.13)	3.91 (1.99, 7.70)	
*Prior MI*					0.4050
No	752	1.0	1.54 (1.06, 2.25)	2.84 (2.01, 4.02)	
Yes	46	1.0	1.09 (0.33, 3.57)	1.20 (0.35, 4.13)	
*Stroke*					0.4004
No	768	1.0	1.52 (1.05, 2.20)	2.75 (1.95, 3.88)	
Yes	30	1.0	0.69 (0.16, 2.91)	0.92 (0.24, 3.58)	
*Clopidogre*					0.4357
No	401	1.0	1.22 (0.75, 1.99)	2.37 (1.55, 3.61)	
Yes	397	1.0	1.97 (1.14, 3.41)	3.04 (1.77, 5.20)	
*Aspirin*					0.4040
No	191	1.0	0.98 (0.47, 2.05)	1.93 (1.08, 3.46)	
Yes	607	1.0	1.73 (1.14, 2.62)	2.85 (1.90, 4.28)	
*Metoprolol*					0.8271
No	236	1.0	1.30 (0.72, 2.35)	1.98 (1.17, 3.35)	
Yes	562	1.0	1.53 (0.97, 2.41)	2.59 (1.68, 4.00)	
*ACEI/ARBs*					0.3793
No	371	1.0	1.33 (0.83, 2.14)	2.21 (1.48, 3.31)	
Yes	427	1.0	1.98 (1.13, 3.47)	2.19 (1.19, 4.03)	
*Statin*					0.2013
No	242	1.0	1.25 (0.73, 2.15)	1.68 (1.04, 2.72)	
Yes	556	1.0	1.67 (1.03, 2.71)	3.08 (1.94, 4.86)	
*PTCA/PCI*					0.2614
No	313	1.0	1.19 (0.71, 1.99)	1.73 (1.10, 2.74)	
Yes	485	1.0	1.69 (1.02, 2.80)	3.08 (1.89, 5.02)	
*Gender*					0.7209
Female	294	1.0	1.37 (0.76, 2.47)	2.82 (1.65, 4.83)	
Male	504	1.0	1.57 (1.00, 2.47)	2.52 (1.65, 3.86)	
*Race*					0.5510
White	518	1.0	1.43 (0.88, 2.34)	2.84 (1.85, 4.38)	
Black	46	1.0	2.63 (0.66, 10.56)	6.29 (1.49, 26.52)	
Other	234	1.0	1.26 (0.70, 2.25)	1.99 (1.14, 3.48)	
*ALT (U/L)*					0.6194
< 35.81	393	1.0	1.35 (0.80, 2.28)	2.88 (1.80, 4.64)	
≥ 35.81	405	1.0	1.57 (0.95, 2.58)	2.41 (1.51, 3.86)	
*AST (U/L)*					0.8455
< 67.00	391	1.0	1.44 (0.82, 2.52)	2.84 (1.71, 4.73)	
≥ 67.00	407	1.0	1.45 (0.91, 2.32) 0	2.40 (1.55, 3.72)	
*CK-peak (U/L)*					0.9398
< 823.00	397	1.0	1.46 (0.88, 2.41)	2.56 (1.64, 4.01)	
≥ 823.00	401	1.0	1.56 (0.93, 2.63)	2.81 (1.71, 4.62)	
*CK-MB-peak (U/L)*					0.9731
< 48.00	398	1.0	1.54 (0.91, 2.60)	2.69 (1.68, 4.30)	
≥ 48.00	399	1.0	1.45 (0.89, 2.38)	2.66 (1.66, 4.28)	
*Glucose (mg/dL*)					0.7956
< 148.00	395	1.0	1.53 (0.89, 2.61)	3.02 (1.85, 4.93)	
≥ 148.00	403	1.0	1.45 (0.89, 2.36)	2.35 (1.50, 3.70)	
*PT (s)*					0.2151
< 13.70	388	1.0	1.96 (1.18, 3.27)	2.82 (1.67, 4.78)	
≥ 13.70	410	1.0	1.03 (0.62, 1.71)	2.00 (1.28, 3.13)	
*Hemoglobin (g/dL)*					0.7119
< 12.40	394	1.0	1.20 (0.71, 2.03)	2.14 (1.34, 3.41)	
≥ 12.40	404	1.0	1.62 (0.98, 2.66)	2.47 (1.47, 4.15)	
*RDW (%)*					0.4510
< 13.80	394	1.0	1.37 (0.77, 2.42)	2.84 (1.65, 4.88)	
≥ 13.80	404	1.0	1.55 (0.97, 2.47)	2.22 (1.45, 3.39)	
*Creatinine (mEq/L)*					0.6118
< 1.10	303	1.0	1.76 (0.91, 3.42)	3.61 (1.91, 6.84)	
≥ 1.10	399	1.0	1.69 (1.05, 2.73)	2.58 (1.66, 4.02)	
*BUN (mg/dL)*					0.1007
< 22.00	370	1.0	2.26 (1.21, 4.21)	3.86 (2.07, 7.20)	
≥ 22.00	414	1.0	1.11 (0.70, 1.75)	1.84 (1.23, 2.77)	
*WBC (10*^*9*^*/L)*					0.3940
< 12.00	383	1.0	1.38 (0.77, 2.46)	3.13 (1.85, 5.29)	
≥ 12.00	401	1.0	1.40 (0.85, 2.31)	2.10 (1.32, 3.35)	
*Potassium (mmol/L)*					0.5270
< 4.20	388	1.0	1.22 (0.71, 2.08)	2.43 (1.48, 3.98)	
≥ 4.20	410	1.0	1.83 (1.13, 2.97)	2.90 (1.85, 4.54)	
*Sodium (mmol/L)*					0.8463
< 139.00	384	1.0	1.64 (0.97, 2.78)	2.91 (1.77, 4.78)	
≥ 139.00	414	1.0	1.35 (0.82, 2.24)	2.46 (1.57, 3.85)	
*Platelet (10*^*9*^*/L)*					0.9992
< 241.00	399	1.0	1.50 (0.90, 2.49)	2.68 (1.70, 4.21)	
≥ 241.00	399	1.0	1.51 (0.91, 2.52)	2.62 (1.61, 4.29)	
*Age (years)*					0.0469
< 70.00	394	1.0	1.26 (0.68, 2.32)	3.42 (1.99, 5.88)	
≥ 70.00	404	1.0	1.58 (1.01, 2.47)	2.05 (1.35, 3.12)	
*Heart rate (beats/minute)*					0.0023
< 101.00	385	1.0	2.61 (1.43, 4.77)	5.12 (2.89, 9.07)	
≥ 101.00	413	1.0	0.92 (0.59, 1.45)	1.51 (1.00, 2.28)	
*Respiratory rate (beats/minute)*					0.8764
< 26.00	368	1.0	1.44 (0.80, 2.58)	2.83 (1.65, 4.83)	
≥ 26.00	430	1.0	1.54 (0.98, 2.44)	2.56 (1.67, 3.90)	
*MBP (mmHg)*					0.4178
< 57.00	371	1.0	1.16 (0.70, 1.92)	2.23 (1.43, 3.46)	
≥ 57.00	427	1.0	1.87 (1.12, 3.13)	2.79 (1.68, 4.66)	
*Weight (kg)*					0.6674
< 78.00	392	1.0	1.76 (1.04, 2.97)	3.01 (1.84, 4.91)	
≥ 78.00	406	1.0	1.26 (0.76, 2.08)	2.32 (1.46, 3.69)	
*ICU LOS (day)*					0.0002
< 3.69	399	1.0	1.28 (0.73, 2.25)	4.33 (2.66, 7.03)	
≥ 3.69	399	1.0	1.40 (0.87, 2.27)	1.59 (1.01, 2.52)	
*SAPS II*					0.0010
< 31.00	247	1.0	8.90 (2.01, 39.44)	19.22 (4.30, 85.91)	
≥ 31.00, < 45	278	1.0	1.10 (0.58, 2.08)	1.33 (0.72, 2.45)	
≥ 45	273	1.0	0.96 (0.60, 1.54)	1.44 (0.95, 2.18)	
*SOFA*					0.0605
< 3.00	247	1.0	2.17 (0.92, 5.11)	5.29 (2.19, 12.78)	
≥ 3.00, < 6.00	236	1.0	1.91 (0.96, 3.82)	1.75 (0.88, 3.50)	
≥ 6.00	315	1.0	0.95 (0.58, 1.54)	1.53 (1.00, 2.33)	
*Urine output (ml/24 h)*					0.2991
< 1805.00	399	1.0	1.26 (0.79, 2.01)	1.84 (1.21, 2.82)	
≥ 1805.00	399	1.0	1.54 (0.87, 2.73)	3.20 (1.86, 5.50)	

*HR* hazard ratio, *CI* confidence interval, *AF* atrial fibrillation, *CHF* chronic heart failure, *CDK* chronic kidney disease, *COPD* chronic obstructive pulmonary disease, *CAD* coronary artery disease, *prior MI* prior myocardial infarction, *ACEI/ARBs* angiotensin-converting enzyme inhibitor/angiotensin receptor blockers, *PTCA/PCI* percutaneous transluminal coronary angioplasty/percutaneous coronary intervention, *ALT* alanine aminotransferase, *AST* aspartate aminotransferase, *CK* creatine kinase, *CK-MB* MB isoenzyme of creatine kinase, *PT* prothrombin time, *RDW* red cell distribution width, *BUN* blood urea nitrogen, *WBC* white blood cell, *MBP* mean blood pressure, *ICU LOS* length of ICU stay, *SAPS II* simplified acute physiology score II, *SOFA* sequential organ failure assessment score

## Discussion

The study indicated that admission NPAR was an independent factor of 30-day, 90-day, 180-day, and 365-day all-cause mortality in critically ill patients with AMI, despite adjustment for age, race and gender. However, after adjustments for more potential confounders, admission NPAR was merely associated with 180-day, and 365-day all-cause mortality. Based on ROC curves, NPAR had a moderate ability to predict all-cause mortality of critically ill patients with AMI. Furthermore, subgroup analysis illustrated that there was no evident interaction in most subgroups.

Inflammation is a hallmark of atherosclerosis where immune cells, especially monocytes and white blood cells, together with cytokines and phospholipids contribute to trigger the inflammatory reaction [5, 15]. The pervious study indicated that neutrophils, as an important member of white blood cells, played a significant role in coronary atherosclerosis and the formation of AMI [16, 17]. Meissner et al. [18] found that high neutrophil count was associated with increased risk for AMI in patients presenting to the emergence department with chest pain. The findings hinted that neutrophil accumulation was a strong predictor of all-cause mortality in patients with AMI [18, 19].

All the time, albumin has been considered as an indicator of nutritional status. However, the current study showed that there was a certain correlation between albumin and inflammation [7, 20, 21]. Serum albumin levels had been affirmed to be inversely related to occurrence of ischemic heart disease [21]. Low serum albumin level was an independent predictor of in-hospital mortality in patients with acute coronary syndrome (ACS) [7, 22]. Furthermore, a significant interaction was found between low serum albumin level and first incident AMI [23] and there was a evident correlation between low serum albumin level long-term mortality in patients with STEMI undergoing PCI [24] and patients with unstable angina pectoris (UAP) or non-ST elevation myocardial infarction (NSTEMI) [25].

The previous studies has demonstrated the prognosis value of NPAR in other clinical events including severe sepsis or septic shock [26], acute kidney injury [27] and cardiogenic shock [28]. Sun et al. [11] documented that NPAR was an independent risk factor in critically ill patients with CAD. In term of the above study, we were curious about that NPAR would have same outcomes in AMI as it did in chronic CAD. The previous study identified that admission NPAR was an independent predictor of in-hospital mortality in patients with STEMI. Based on this, we investigated the impact of admission NPAR on the short-term and long-term risk of death in AMI patients. Our study showed that higher admission NPAR was only associated with increased risks of 180-day and 365-day all-cause mortality and may be an independent marker for long-term all-cause mortality in critically ill patients with AMI. According to the AUC area, admission NPAR had a moderate predictive ability in critically ill patients with AMI.

Compared with SOFA and SAPS II, the capacity of admission NPAR in predicting the risk of death was as well as SOFA though its effectiveness was not so adequate than SAPS II. However, by comparing SOFA score with the integrated model, we could speculate that admission NPAR was added to SOFA score may played a guiding role in predicting the all-cause mortality.

On account of the test methods for neutrophil percentage and albumin are economical and practical, admission NPAR would permit it possible to quickly evaluate the risk of death in critically ill patients with AMI. Especially, it has precious clinical utility for areas with underdeveloped economy and poor medical conditions.

## Limitation

This study was a single-center retrospective study, the selection bias was inevitable. All data came from a publicly open clinical database, so it was difficult to extract some important variables, such as the history of smoking and drinking. Since both neutrophil percentage and albumin change dynamically, the study just chose admission NPAR measured at the first time after admission. Random error maybe inevitable. Due to missing values of more than 20%, it was difficult to obtain information about some important clinical or laboratory variables.

## Conclusion

Our study suggests that higher admission NPAR was independently associated with 180-day and 365-day all-cause mortality in critically ill patients with AMI. NPAR may be a clinical maker to predict risk stratification in patients with AMI and further to offer the Individualized treatment services.

## Data Availability

The datasets generated and analysed during the current study are available in the [Medical Information Mart for Intensive Care III (MIMIC III)] repository, [https://physionet.org/content/mimiciii/1.4/].

## Abbreviations

ACEI: Angiotensin-converting enzyme inhibitor
ACS: Acute coronary syndrome
AF: Atrial fibrillation
ALT: Alanine aminotransferase
AMI: Acute myocardial infarction
ARB: Angiotensin receptor blocker
AST: Aspartate aminotransferase
AUC: Area under the curve
BUN: Blood urea nitrogen
CAD: Coronary artery disease
CDK: Chronic kidney disease
CHF: Chronic heart failure
CI: Confidence interval
CK: Creatine kinase-peak
CK-MB: MB isoenzyme of creatine kinase
COPD: Chronic obstructive pulmonary disease
HR: Hazard ratios
ICU: Intensive care units
IDI: Integrated discrimination improvement
IRB: Institutional Review Boards
LOS: Length of stay
MBP: Mean blood pressure
MI: Myocardial infarction
MIMIC III: Medical Information Mart for Intensive Care III
MIT: Massachusetts Institute of Technology
NIH: National Institutes of Health
NPAR: Neutrophil percentage-to-albumin ratio
NSTEMI: Non-ST elevation myocardial infarction
PCI: Percutaneous coronary intervention
PT: Prothrombin time
PTCA: Percutaneous transluminal coronary angioplasty
RDW: Red cell distribution width
ROC: Receiver-operating characteristic
SAPS II: Simplified acute physiology score II
SOFA: Sequential organ failure assessment
SQL: Structured Query Language
STEMI: ST-segment elevation myocardial infarction
UAP: Unstable angina pectoris
WBC: White blood cell

## References

[1] Reed GW, Rossi JE, Cannon CP (2017). Acute myocardial infarction. Lancet.

[2] Suh JW, Mehran R, Claessen BE, Xu K, Baber U, Dangas G (2011). Impact of in-hospital major bleeding on late clinical outcomes after primary percutaneous coronary intervention in acute myocardial infarction the HORIZONS-AMI (Harmonizing Outcomes With Revascularization and Stents in Acute Myocardial Infarction) trial. J Am Coll Cardiol.

[3] Ong SB, Hernandez-Resendiz S, Crespo-Avilan GE, Mukhametshina RT, Kwek XY, Cabrera-Fuentes HA (2018). Inflammation following acute myocardial infarction: multiple players, dynamic roles, and novel therapeutic opportunities. Pharmacol Ther.

[4] Granger CB, Kochar A (2018). Understanding and targeting inflammation in acute myocardial infarction: an elusive goal. J Am Coll Cardiol.

[5] Ruparelia N, Chai JT, Fisher EA, Choudhury RP (2017). Inflammatory processes in cardiovascular disease: a route to targeted therapies. Nat Rev Cardiol.

[6] Roche M, Rondeau P, Singh NR, Tarnus E, Bourdon E (2008). The antioxidant properties of serum albumin. FEBS Lett.

[7] Gonzalez-Pacheco H, Amezcua-Guerra LM, Sandoval J, Martinez-Sanchez C, Ortiz-Leon XA, Pena-Cabral MA (2017). Prognostic implications of serum albumin levels in patients with acute coronary syndromes. Am J Cardiol.

[8] Wada H, Dohi T, Miyauchi K, Shitara J, Endo H, Doi S (2017). Impact of serum albumin levels on long-term outcomes in patients undergoing percutaneous coronary intervention. Heart Vessels.

[9] Plakht Y, Gilutz H, Shiyovich A (2016). Decreased admission serum albumin level is an independent predictor of long-term mortality in hospital survivors of acute myocardial infarction. Soroka Acute Myocardial Infarction II (SAMI-II) project. Int J Cardiol.

[10] Cui HH, Ding XS, Li WP, Chen H, Li HW, Data CBFH (2019). The neutrophil percentage to albumin ratio as a new predictor of in-hospital mortality in patients with ST-segment elevation myocardial infarction. Med Sci Monitor.

[11] Sun TN, Shen H, Guo QY, Yang JQ, Zhai GY, Zhang JR, et al. Association between neutrophil percentage-to-albumin ratio and all-cause mortality in critically ill patients with coronary artery disease. Biomed Res Int. 2020;2020. 10.1155/2020/8137576 PMC7479485 32934964

[12] Johnson AE, Pollard TJ, Shen L, Lehman LW, Feng M, Ghassemi M (2016). MIMIC-III, a freely accessible critical care database. Sci Data.

[13] Ferreira FL, Bota DP, Bross A, Melot C, Vincent JL (2001). Serial evaluation of the SOFA score to predict outcome in critically ill patients. JAMA.

[14] Le Gall JR, Lemeshow S, Saulnier F (1993). A new Simplified Acute Physiology Score (SAPS II) based on a European/North American multicenter study. JAMA.

[15] Hansson GK (2005). Inflammation, atherosclerosis, and coronary artery disease. N Engl J Med.

[16] Chistiakov DA, Grechko AV, Myasoedova VA, Melnichenko AA, Orekhov AN (2018). The role of monocytosis and neutrophilia in atherosclerosis. J Cell Mol Med.

[17] Silvestre-Roig C, Braster Q, Ortega-Gomez A, Soehnlein O (2020). Neutrophils as regulators of cardiovascular inflammation. Nat Rev Cardiol.

[18] Meissner J, Irfan A, Twerenbold R, Mueller S, Reiter M, Haaf P (2011). Use of neutrophil count in early diagnosis and risk stratification of AMI. Am J Med.

[19] Kyne L, Hausdorff JM, Knight E, Dukas L, Azhar G, Wei JY (2000). Neutrophilia and congestive heart failure after acute myocardial infarction. Am Heart J.

[20] Don BR, Kaysen G (2004). Serum albumin: relationship to inflammation and nutrition. Semin Dial.

[21] Arques S (2018). Human serum albumin in cardiovascular diseases. Eur J Intern Med.

[22] Zhu L, Chen M, Lin X (2020). Serum albumin level for prediction of all-cause mortality in acute coronary syndrome patients: a meta-analysis. Biosci Rep.

[23] He YM, Yang Q, Yang XJ, Zhao X, Xu HF, Qian YX (2016). Serum albumin concentrations, effect modifiers and first incident acute myocardial infarction: a cross-sectional study of 1552 cases and 6680 controls. Clin Chim Acta.

[24] Oduncu V, Erkol A, Karabay CY, Kurt M, Akgun T, Bulut M (2013). The prognostic value of serum albumin levels on admission in patients with acute ST-segment elevation myocardial infarction undergoing a primary percutaneous coronary intervention. Coron Artery Dis.

[25] Polat N, Oylumlu M, Isik MA, Arslan B, Ozbek M, Demir M (2020). Prognostic significance of serum albumin in patients with acute coronary syndrome. Angiology.

[26] Gong YQ, Li DW, Cheng BH, Ying BY, Wang BJ (2020). Increased neutrophil percentage-to-albumin ratio is associated with all-cause mortality in patients with severe sepsis or septic shock. Epidemiol Infect.

[27] Wang BJ, Li DW, Cheng BH, Ying BY, Gong YQ (2020). The neutrophil percentage-to-albumin ratio is associated with all-cause mortality in critically ill patients with acute kidney injury. Biomed Res Int.

[28] Yu Y, Liu Y, Ling XY, Huang RH, Wang SY, Min J, et al. The neutrophil percentage-to-albumin ratio as a new predictor of all-cause mortality in patients with cardiogenic shock. Biomed Res Int. 2020;2020. 10.1155/2020/7458451 PMC7714577 33294452

